# Safety and clinical efficacy of immune checkpoint inhibitors in advanced gastric cancer in the real world

**DOI:** 10.1007/s00432-024-05703-8

**Published:** 2024-04-08

**Authors:** Wen Hao, Wenjing Liu, Ruimin Chang, Mi Yang, Kai Xin, Jingxin Liu, Yibing Wang, Meijin Ren, Jiaqi Xie, Yang Yang

**Affiliations:** 1 Department of Oncology, Nanjing Drum Tower Hospital, Drum Tower Hospital Clinical College, Nanjing University of Chinese Medicine, Nanjing, China; 2 Nanjing Drum Tower Hospital, Affiliated Hospital of Medical School, Nanjing University, Nanjing, China

**Keywords:** Gastric cancer, Immune checkpoint inhibitor, Efficacy, Multivariate analysis, Adverse effects

## Abstract

**Background:**

To evaluate the clinical efficacy and safety of immune checkpoint inhibitors in patients with advanced gastric cancer in the real world.

**Methods:**

The retrospective analysis was conducted on the clinical records of 402 patients with advanced gastric cancer who were admitted to the Nanjing Drum Tower Hospital between December 2017 and April 2022 and who had received immunotherapy. Observation target: drug use, treatment, adverse reaction type and grade, objective response rate (ORR), disease control rate (DCR), progression free survival (PFS), and overall survival (OS).

**Results:**

By retrospectively analyzing the data of patients with advanced gastric cancer treated with ICIs previously admitted to our medical center, we found some clinical characteristic factors associated with the occurrence of irAEs as well as the efficacy and prognosis: the presence or absence of hypertension, whether or not to receive targeted therapies can predict the occurrence of immune-related adverse events (irAEs), and the more the presence of irAEs, the better the prognosis. These can help clinicians in clinical drug selection.

**Conclusions:**

The results of this paper show that the occurrence of irAEs is associated with patients’ OS. irAEs occurrence can prolong patients’ OS. irAEs occurrence may serve as a surrogate marker for ICIs.

**Supplementary Information:**

The online version contains supplementary material available at 10.1007/s00432-024-05703-8.

## Introduction

According to the data related to 2020, gastric cancer is the fifth most common cancer worldwide and the fourth most common cause of cancer deaths (Sung et al. 2021). China has one of the highest incidence rates of gastric cancer (Smyth et al. 2020). Palliative chemotherapy is the corner-stone of treatment for patients with advanced gastric cancer who have a poor prognosis with a median overall survival of 10 to 12 months (Digklia and Wagner 2016). However, the success of immune checkpoint therapies targeting cytotoxic T-lymphoid-associated protein-4 (CTLA-4), programmed death receptor-1 (PD-1), and programmed death-ligand 1 (PD-L1) has led to a breakthrough in tumor immunotherapy (Li et al. 2019), numerous clinical trials have also successfully revealed that PD-1/PD-L1 immune checkpoint inhibitors exhibit favorable therapeutic effects (Wang et al. 2024).

Relevant trials on immunotherapy have been conducted in the clinic, confirming that immunotherapy for gastric cancer has excellent therapeutic prospects. One is based on the ATTRACTION-2 trial, in which nivolumab has been approved in Japan for the third-line treatment of recurrent or metastatic adenocarcinoma of the stomach or gastroesophageal–geal junction (Allemani et al. 2018). Another anti-PD-1 monoclonal antibody, pembrolizumab, was previously approved by the US Food and Drug Administration (FDA) for the treatment of gastric cancer (PD-L1-positive) in the third or backline, based on the results of a significant phase II trial (Kawazoe et al. 2021). In addition, based on the results of the CheckMate 649, the first-line trial showed that nivolumab, in combination with the chemotherapy group, also better prolonged OS and PFS in patients with a CPS of 5 (Moehler et al. 2020). All these indicate that this immunotherapy is promising and will bring new hope to patients with advanced gastric cancer.

Rashes, colitis, hepatitis, hypo-or hyperthyroidism, and pneumonia are common irAEs (Friedman et al. 2016). The prevalence of irAEs worldwide may differ due to various immunotherapy studies, and there needs to be more epidemiologic data on irAEs and other influencing factors in China. The study examined the clinical features, incidence, treatment approaches, and the effectiveness and Prognosis of ICIs in actual adverse events (irAEs) in the Chinese population.

## Materials and methods

### Clinical information

This work presents a real-world retrospective analysis of patients with advanced gastric cancer who were treated with applied PD-1 inhibitors and were admitted to the Oncology Center of Nanjing Drum Tower Hospital between December 2017 and April 2022. Patient clinical information was gathered: (1) Demographics: age, gender, BMI, comorbidities, history of alcohol usage, history of smoking, and ECOG score. (2) The characteristics of the tumor include its stage, degree of differentiation, WHO and Lauren classification, number of metastases, and place of metastasis. (3) Treatment: whether they had gastrectomy, previous treatments, previous treatment modalities, immunotherapy drugs used, dose of medicine immunotherapy modalities, immune combination modalities, and the number of patients with advanced gastric cancer treated with PD-1 inhibitors. Immunotherapeutic drugs, immunotherapy combination therapy, tumor response characteristics, number of immunotherapy treatment lines. (4) Lab examinations: (i) immunohistochemistry: tumor mutation burden (TMB), microsatellite (MS), Ki67 index, human epidermal growth factor receptor-2 (HER2), vascular endothelial growth factor receptor-2, (VEGFR2), mismatch repair (MMR), epithelial-cadherin (E-cad), and PD-L1. (ii) Tumor markers (2 months post-dose and 1-week pre-dose): alpha-fetoprotein (AFP), carcinoembryonic antigen (CEA), carbohydrate antigen199 (CA199), carbohydrate antigen242 (CA242). (iii) Liver function indicators (ratio of 2 months prior to medication and 1 week prior to medication): alanine aminotransferase (ALT), aspartate transaminase (AST). In our database, the first-line regimen for immunotherapy for advanced gastric cancer mainly used a combination of PD-1 monoclonal antibodies with Capecitabine and Oxaliplatin (CAPOX) or tegafur gimeracil oteracil potassium capsule plus oxaliplatin (SOX) regimens. For second-line treatment, regimens of PD-1 monoclonal antibody combined with paclitaxel analogs or camptothecin analogs were selected. Third-line treatment included the use of PD-1 monoclonal antibody combined with apatinib or other targeted drugs. The above treatment plan is formulated by clinicians using the Chinese Society of Clinical Oncology Guidelines for Gastric Cancer Diagnosis and Treatment as a benchmark and integrating their rich clinical experience. For patient-specific medication information, refer to Table I of the supplemental material.

### Toxicological classification

The management of toxicity grading of irAEs is currently typically referenced to the CTCAE V5.0: while grade 1 is mild and doesn't require treatment, grade 2 is moderate and calls for either localized treatment or an outpatient course of oral or local glucocorticoid treatment; grade 3 is severe or medically significant, but not life-threatening, requiring hospitalization with systemic glucocorticoid therapy and a decision about whether to continue immunotherapy based on the risk assessment; grade 4 is life-threatening and necessitates either immediate treatment or a permanent stop to immunotherapy along with concurrent use of glucocorticoids and other immunosuppressive agents; and grade 5 refers to irAEs-related deaths (Freites-Martinez et al. 2021).

### Evaluations and criteria

According to the solid tumor efficacy evaluation standard RECIST version 1.1 efficacy evaluation was divided into complete response (CR), partial response (PR), stable disease (SD), and progressive disease (PD); the objective response rate (ORR) was defined as (CR + PR)/number of evaluable cases × 100%, while the disease control rate (DCR) was defined as (CR + PR + SD)/number of evaluable cases × 100% (Eisenhauer et al. 2009), PFS was defined as the time from the initial administration of immune checkpoint inhibitors to tumor progression or death, and PFS was calculated at the cutoff point of the last follow-up (October 19, 2022) for nonprogressing patients; OS was defined as the time from the initial administration of immune checkpoint inhibitors to the death of the patients, and survival was calculated as the cutoff time of October 19, 2022, for nonprogressing patients.

### Statistical methods

Drawing with GraphPad software, the SPSS 26.0 software was used for statistical analysis, and the normal distribution of quantitative data was tested; if it conformed to the normal distribution, it was expressed as $\overline{x} \pm s$, and the t test was used for between-group comparison; if it did not work to the normal distribution, it was described as M (Q1, Q3) and the Mann–Whitney *U* test was used for between-group comparison. Qualitative data were described as frequency (percentage) and analyzed by *χ*^2^ test or Fisher’s exact test. The related factors were analyzed by univariate and multifactorial logistic regression analysis, Kaplan–Meier survival curves were plotted, and the relationship between each factor and the observed indexes OS and PFS was analyzed using COX regression. (Statistically significant when *P* < 0.05).

## Results

### Patient grouping and clinical data analysis

402 patients with advanced gastric cancer who received immunotherapy were included, and 191 (47.51%) cases occurred with different types and degrees of irAEs, with or without hypertension (*P* = 0.008), with or without diabetes mellitus (*P* = 0.048); the difference between count groups was statistically significant (*P* < 0.05) (Table 1).

**Table 1:** Patient grouping and clinical data analysis

Clinical variables	irAEs group (*n* = 191)	Non-irAEs group (*n* = 211)	*P*
Age	63.0 (54.69)	63.0 (55.69)	0.686
BMI	22.4 ± 3.158	22.7 ± 3.208	0.491
Hypertension			0.008
Yes	60 (31.4)	42 (19.9)	
No	131 (68.6)	169 (80.1)	
Hepatitis			0.886
Yes	12 (6.2)	14 (6.6)	
No	179 (93.8)	197 (93.4)	
Coronary heart disease			1.000
Yes	4 (2.0)	4 (1.9)	
No	187 (98.0)	207 (98.1)	
Diabetes			0.048
Yes	26 (13.6)	16 (7.6)	
No	165 (86.4)	195 (92.4)	
Genders			0.622
Male	140 (73.0)	150 (71.0)	
Female	51 (27.0)	61 (19.0)	
Pathological classification			0.512
Adenocarcinoma	163 (85.3)	176 (83.4)	
Signet-ring cell carcinoma	17 (8.9)	25 (11.9)	
Others	11 (5.8)	10 (4.7)	
TNM staging			0.501
Stage III	13 (6.8)	11 (5.2)	
Stage IV	178 (93.2)	200 (94.8)	
Site of the primary tumor			0.942
Proximal stomach	56 (29.3)	64 (30.3)	
Distal stomach	105 (55.0)	117 (55.5)	
Others	30 (15.7)	30 (14.2)	
Peritoneal metastasis			0.491
Yes	75 (39.2)	90 (42.7)	
No	116 (60.8)	121 (57.3)	
Liver metastasis			0.370
Yes	65 (34.0)	63 (30.0)	
No	126 (66.0)	148 (70.0)	
Bone metastasis			0.393
Yes	16 (8.4)	23 (10.9)	
No	175 (91.6)	188 (89.1)	
Pulmonary metastasis			0.150
Yes	6 (3.1)	13 (6.2)	
No	185 (96.9)	198 (93.8)	
Retroperitoneal lymph node metastasis			0.209
Yes	34 (17.8)	28 (13.3)	
No	157 (18.2)	183 (86.7)	
Number of transfers			0.905
≤ 2	178 (93.2)	196 (92.9)	
≥ 3	13 (6.8)	15 (7.1)	
Number of immunotherapy treatment lines			0.854
First line	170 (89.0)	189 (89.6)	
Second line and above	21 (11.0)	22 (10.4)	
Gastrectomy			0.096
Yes	89 (46.5)	81 (38.4)	
No	102 (53.5)	130 (61.6)	
Previous chemotherapy			0.985
Yes	65 (34.0)	72 (34.1)	
No	126 (66.0)	139 (65.9)	
Previous targeted therapy			0.063
Yes	7 (3.7)	17 (8.0)	
No	184 (96.3)	194 (92.0)	
Liver radiotherapy			0.839
Yes	8 (4.2)	8 (3.8)	
No	183 (95.8)	203 (96.2)	
Immunotherapy combined with chemotherapy			0.861
Yes	165 (86.4)	181 (85.8)	
No	26 (13.6)	30 (14.2)	
Immunotherapy combined with radiotherapy			0.090
Yes	35 (18.3)	26 (12.3)	
No	156 (81.7)	185 (87.7)	
Immunotherapy combined with targeted therapy			0.170
Yes	43 (22.5)	36 (17.1)	
No	148 (77.5)	175 (82.9)	
KI67^a^			0.932
< 30%	13 (13.8)	11 (14.3)	
≥ 30%	81 (86.2)	66 (85.7)	
VEGFR2^a^			0.281
Negative	27 (39.7)	19 (30.6)	
Positive	41 (60.3)	43 (69.4)	
E-cadherin^a^			0.585
Negative	9 (10.1)	6 (7.7)	
Positive	80 (89.9)	72 (92.3)	
MMR/microsatellite^a^			0.529
dMMR/MSI-H	12 (8.0)	9 (6.4)	
pMMR/MSS	131 (92.0)	131 (93.6)	
Smoking history			0.706
Yes	40 (20.9)	41 (19.4)	
No	151 (79.1)	170 (80.6)	
Drinking history			0.623
Yes	30 (15.7)	37 (17.5)	
No	161 (84.3)	174 (82.5)	
ECOG scores			0.093
0–1	172 (90.0)	191 (94.6)	
≥ 2	19 (10.0)	11 (5.4)	
Lauren classification^a^			0.339
Intestinal	34 (47.9)	28 (40.0)	
Diffuse	21 (29.6)	29 (41.4)	
Mixed	16 (22.5)	13 (18.6)	
Differentiated			0.827
Poorly differentiated	144 (75.4)	155 (73.5)	
Moderately differentiated	30 (15.7)	38 (18.0)	
Others	17 (8.9)	18 (8.5)	
Type of PD-1			0.301
Nivolumab	31 (15.5)	52 (23.7)	
Sindilizumab	63 (31.5)	62 (28.3)	
Tislelizumab	44 (22.0)	40 (18.3)	
Treprinumab	43 (21.5)	47 (21.5)	
Others	19 (9.5)	18 (8.2)	
HER2^a^			0.480
Negative	111 (79.3)	103 (75.7)	
Positive	29 (20.7)	33 (24.3)	
PD-L1 (SP162)^a^			0.501
< 1%	20 (19.8)	28 (26.7)	
≥ 1–5%	41 (40.6)	38 (36.2)	
> 5%	40 (39.6)	39 (37.1)	
CA199 ratio (2 months after medication/1 week before medication)			0.818
Both normal or ratio ≤ 0.75	70 (36.6)	75 (35.5)	
Ratio > 0.75	121 (63.4)	136 (64.5)	
CEA ratio (2 months after medication/1 week before medication)			0.389
Both normal or ratio ≤ 0.75	74 (38.7)	73 (34.6)	
Ratio > 0.75	117 (61.3)	138 (65.4)	
AFP ratio (2 months after medication/1 week before medication)			0.627
Both normal or ratio ≤ 0.75	65 (34.0)	67 (31.8)	
Ratio > 0.75	126 (66.0)	144 (68.2)	
CA242 ratio (2 months after medication/1 week before medication)			0.498
Both normal or ratio ≤ 0.75	76 (39.8)	91 (43.1)	
Ratio > 0.75	115 (60.2)	120 (56.9)	
ALT ratio (2 months after medication/1 week before medication)			0.273
Both normal or ratio ≤ 0.75	30 (15.7)	42 (19.9)	
Ratio > 0.75	161 (84.3)	169 (80.1)	
AST ratio (2 months after medication/1 week before medication)			0.052
Both normal or ratio ≤ 0.75	25 (13.1)	43 (20.4)	
Ratio > 0.75	166 (86.9)	168 (79.6)	

^a^Data were missing due to the fact that immunohistochemistry was not performed on some patients at our institution or was performed at an outside institution, as well as incomplete patient information

### Types of irAEs in patients

**Fig. 1:**
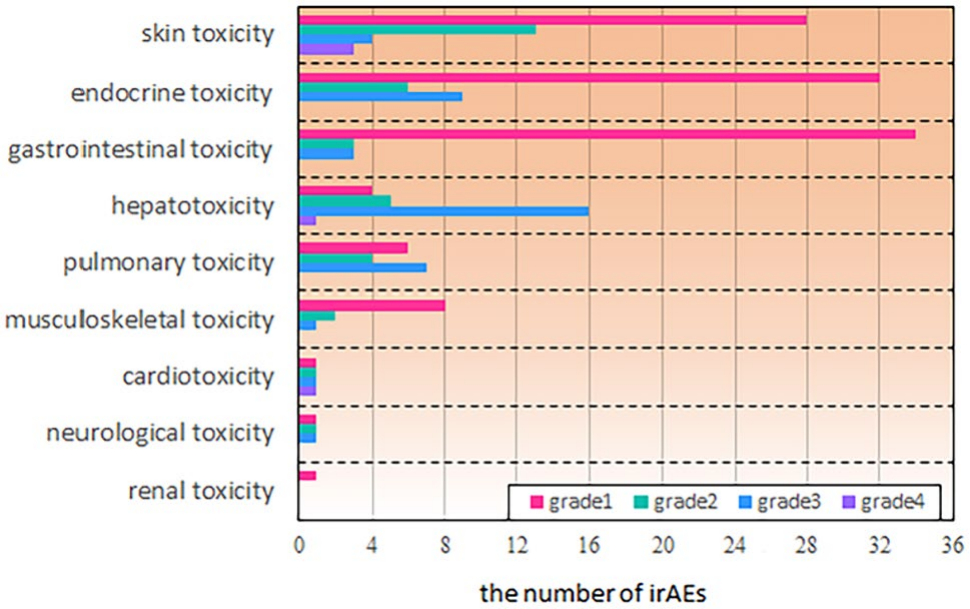
Type and toxicity level distribution of irAEs in patients

Data analysis of irAEs revealed skin toxicity in 48 cases (24.4%), endocrine toxicity in 47 cases (23.9%), gastrointestinal toxicity in 40 cases (20.3%), hepatotoxicity in 26 cases (13.2%), pulmonary toxicity in 17 cases (8.6%), musculoskeletal toxicity in 11 cases (5.6%), cardiotoxicity in 4 cases (2.0%), neurological toxicity in 3 cases (1.5%), and neurological toxicity in 1 case (0.5%). The highest incidence was found in skin rash (skin toxicity), nail function abnormality (endocrine toxicity), and diarrhea (gastrointestinal toxicity), with 55 (28.8%) of the 197 cases discontinuing treatment due to irAEs and 1 case death due to immune-associated pneumonia. When hormone therapy is used in clinical practice, the occurrence of irAEs was mainly concentrated in grades 1 and 2 (Fig. 1).

### Distribution of time to onset of irAEs in patients

**Fig. 2:**
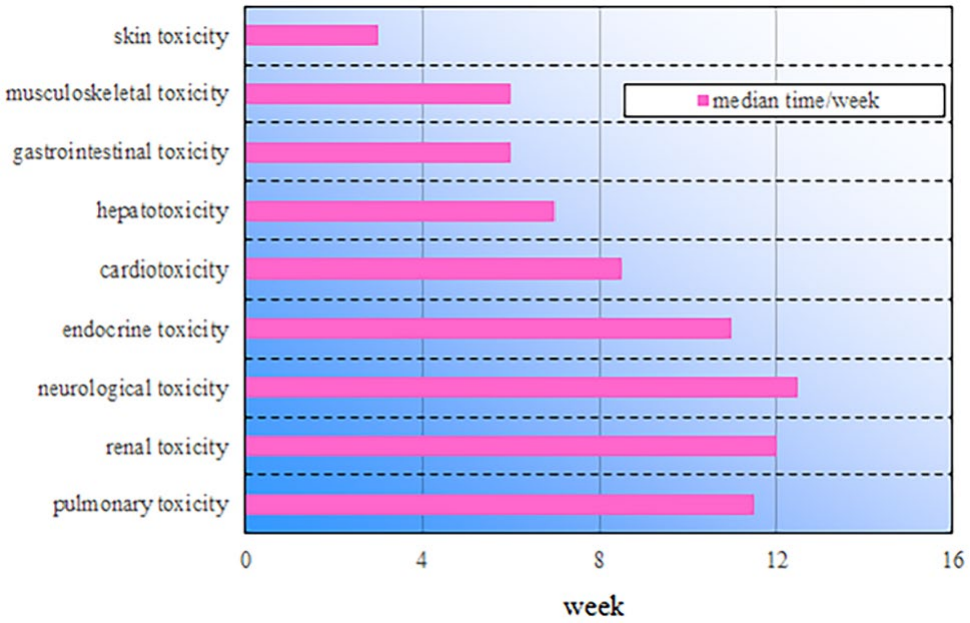
Median time distribution of patient irAEs

We performed an in-depth analysis of the data on all irAEs that occurred in patients after receiving immunotherapy up to the study cut-off date. It was observed that the longest immune-related irAE occurred at week 30 after the start of immunotherapy, whereas the median onset time of all irAEs and their range was approximately 7 (1–30) weeks. Specifically, the median onset times of 12 (12–12) weeks for immune-related renal toxicity, 11.5 (5–20) weeks for immune-related pulmonary toxicity, 8.5 (7–10) weeks for immune-related cardiotoxicity, 11 (1–30) weeks for immune-related endocrine toxicity, and 12.5 (12–13) weeks for immune-related neurological toxicity were around 12 weeks. On the other hand, the median onset times of immune-related skin toxicity were 3 (1–12) weeks, immune-associated gastrointestinal toxicity was 6 (1–12) weeks, immune-associated hepatotoxicity was 7 (4–18) weeks, and immune-associated musculoskeletal toxicity was 6 (2–15) weeks. These data clearly reveal the temporal distribution of different types of immune-related adverse events in patients receiving immunotherapy up to the study cutoff date (Fig. 2).

### Immune-related toxicity brought on by several PD-1

Among the 402 patients, the use of immune checkpoint inhibitors included 121 cases of sintilimab, 90 cases of toripalimab, 84 cases of tislelizumab, 83 cases of nivolumab, 16 cases of camrelizumab, 13 cases of pembrolizumab), and 6 cases of penpulimab. The use of immune checkpoint inhibitors in patients with irAEs included 68 cases (56.2%) of sintilimab, 45 cases (50.0%) of toripalimab, 42 cases (50.0%) of tislelizumab, 32 cases (38.6%) of nivolumab, 11 cases (68.8%) of camrelizumab, 5 cases (38.5%) of pembrolizumab, and 2 cases (33.3%) of penpulimab. These seven PD-1s most frequently showed skin toxicity, endocrine toxicity, and gastrointestinal toxicity (Table 2).

**Table 2:** Immune-related toxicity brought on by several PD-1

irAEs	Sintilimab *n* (%)	Toripalimab *n* (%)	Tislelizumab *n* (%)	Nivolumab *n* (%)	Camrelizumab *n* (%)	Pembrolizumab *n* (%)	Penpulimab *n* (%)
Aggregate	68 (56.2)	45 (50.0)	42 (50.0)	32 (38.6)	11 (68.8)	5 (38.5)	2 (33.3)
Skin toxicity	18 (14.9)	11 (12.2)	13 (15.5)	4 (4.8)	3 (18.8)	1 (7.7)	1 (16.7)
Hepatotoxicity	7 (5.8)	6 (6.7)	4 (4.8)	7 (8.4)	1 (6.3)	0 (0.0)	1 (16.7)
Gastrointestinal Toxicity	14 (11.6)	11 (12.2)	8 (9.5)	6 (7.2)	2 (12.5)	1 (7.7)	0 (0.0)
Pulmonary toxicity	6 (5.0)	0 (0.0)	4 (4.8)	5 (6.0)	0 (0.0)	0 (0.0)	0 (0.0)
Musculoskeletal toxicity	7 (5.8)	1 (1.1)	1 (1.2)	0 (0.0)	0 (0.0)	0 (0.0)	0 (0.0)
Renal toxicity	0 (0.0)	0 (0.0)	0 (0.0)	0 (0.0)	0 (0.0)	1 (7.7)	0 (0.0)
Endocrine toxicity	16 (13.2)	15 (16.7)	10 (11.9)	8 (9.6)	4 (25.0)	2 (15.4)	0 (0.0)
Cardiotoxicity	0 (0.0)	1 (1.1)	1 (1.2)	2 (2.4)	0 (0.0)	0 (0.0)	0 (0.0)
Neurological toxicity	0 (0.0)	0 (0.0)	1 (1.2)	0 (0.0)	1 (6.3)	0 (0.0)	0 (0.0)

### Logistic regression of factors associated with patients’ irAEs

Logistic univariate regression analysis showed that Lauren classification (OR: 0.284, 95% CI 0.111–0.726), the presence of hypertension (OR: 1.843, 95% CI 1.169–2.906), whether or not they had received prior chemotherapy (OR: 1.805, 95% CI 1.059–3.078), whether or not they had received prior targeted therapy (OR: 2.000, 95% CI 1.429–3.024) were statistically significant (*P* < 0.05). Variables that were different in an one-way logistic regression analysis of Tables 1 and 2 were included in the logistic.

Multifactorial regression analysis (the multivariate logistic regression analysis does not include the presence or absence of diabetes and previous chemotherapy since they are collinearly connected with hypertension, previous chemotherapy, and previous targeted treatment). Multifactorial logistic regression analysis showed that having hypertension (OR: 2.759, 95% CI 1.206–6.310) and receiving targeted therapy (OR: 4.360, 95% CI 1.692–11.238) were the risk factors influencing the irAEs (*P* < 0.05) (Table 3).

**Table 3:** Univariate and multivariate Logistic regression analysis for irAEs

Clinical variables	Univariate logistic analysis		Multivariate logistic analysis	
	OR (95% CI)	*P*	OR (95% CI)	*P*
Lauren classification				
Intestinal	1		1	
Diffuse	0.684 (0.322–1.455)	0.324	0.782 (0.349–1.751)	0.549
Mixed	0.284 (0.111–0.726)	0.009	0.376 (0.139–1.017)	0.054
Hypertension				
No	1		1	
Yes	1.843 (1.169–2.906)	0.009	2.759 (1.206–6.310)	0.016
Diabetes				
No	1			
Yes	1.920 (0.976–3.702)	0.051		
Previous chemotherapy				
No	1			
Yes	1.805 (1.059–3.078)	0.03		
Previous targeted therapy				
No	1		1	
Yes	2.000 (1.429–3.024)	0.004	4.360 (1.692–11.238)	0.002

### Efficacy evaluation

#### Patient efficacy evaluation and prognosis

**Fig. 3:**
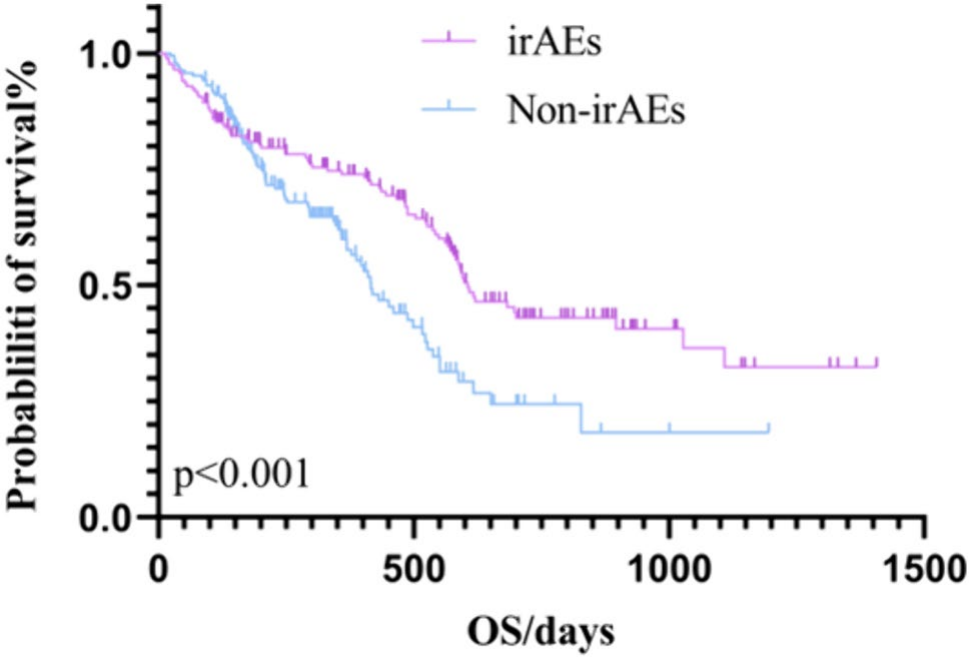
Kaplan–Meier survival curve for first-line OS with immunotherapy

**Fig. 4:**
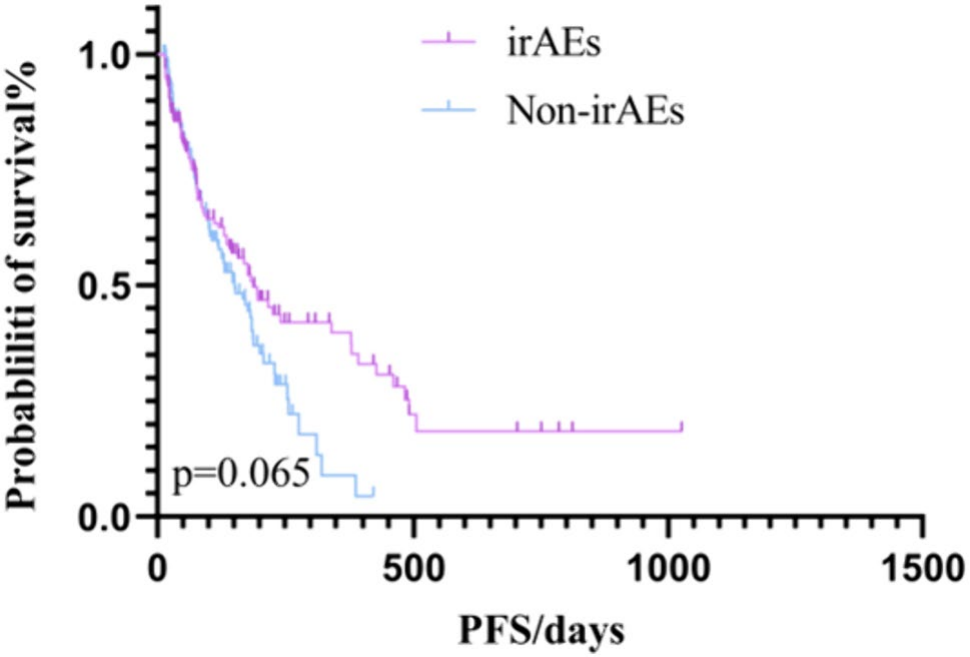
Kaplan–Meier survival curve for first-line PFS with immunotherapy

Tumor efficacy evaluation criteria were adopted from RECIST version 1.1, and the analysis results showed that, in the first-line treatment of immunity, the number of evaluable cases in irAEs group after 2 months of drug administration was 170, with 4 (2.3%) patients CR, 53 (31.1%) PR, 83 (48.8%) SD., and 30 (17.8%) PD, with an ORR of 33.5%, and a DCR of 82.3%. The number of evaluable cases after 2 months of drug administration in the non-irAEs group was 189, with 4 (2.1%) patients with CR, 62 (32.8%) with PR, 83 (43.9%) with SD, and 40 (21.2%) with PD, with an ORR of 34.9%, and a DCR of 78.8% (Table 4). (21 patients in the backline irAEs group and 22 patients in the non-irAEs group in the immunotherapy group; the sample data is too small and the data may be in error, so backline patients are not discussed). Kaplan–Meier survival curves showed that the mPFS in the immunotherapy first-line irAEs group was188 days (95% CI 136.193–239.807) and the mOS was 605 days (95% CI 516.492–693.510). The mPFS in the immunotherapy first-line non-irAEs group was 152 days (95% CI 111.733–192.227), and the mOS was 416 days (95% CI 357.866–474.136) (Figs. 3 and 4).

**Table 4:** Evaluation of the efficacy of first-line immunotherapy in patients

Therapeutic effect	irAEs group *n* (%)	Non-irAEs group *n* (%)
CR	4 (2.3)	4 (2.1)
PR	53 (31.1)	62 (32.8)
SD	83 (48.8)	83 (43.9)
PD	30 (17.8)	40 (21.2)
ORR	57/170 (33.5)	66/189 (34.9)
DCR	140/170 (82.3)	149/189 (78.8)

#### Analysis of patient prognostic OS-related influencing factors

The case characteristic variables in Table 1 were added to one-way COX regression, and the results showed that the risk of death was higher in the control group for ECOG score ≥ 2 (HR: 2.700, 95% CI 1.528–4.770), with liver metastasis (HR: 5.351, 95% CI 3.976–7.202), with hypertension (HR: 0.594, 95% CI 0.418–0.844), adverse reactions occurred (HR: 0.669, 95% CI 0.498–0.900), ALT (ratio of 2 months after dosing to 1 week before dosing) > 0.75 (HR: 0.621, 95% CI 0.434–0.888), AST (ratio of 2 months after dosing to 1 week before dosing) > 0.75 (HR: 0.546, 95% CI 0.380–0.786), lower risk of death as compared to controls.

Variables with *P* values of 0.05 or less on univariate testing (ALT was excluded from the multivariate logistic regression analysis due to the presence of a covariate relationship between ALT and AST) were attempted to be added to the multivariate regression analysis corrected for the presence of liver metastases (HR: 6.110 95% CI 3.354–11.131) with ECOG scores of ≥ 2 (HR: 1.885, 95% CI 1.061–3.347) had a higher risk of death than the control group, which was still negatively correlated with the prolongation of OS, and the risk of death of hypertension (HR: 0.680, 95% CI 0.474–0.973) and irAEs (HR: 0.606, 95% CI 0.444–0.827) was lower than the control group, which was still positively correlated with the prolongation of OS (Table 5).

**Table 5:** Univariate and multivariate COX regression analysis of OS

OS	Univariate Cox analysis		Multivariate Cox analysis	
Clinical variables	HR (95% CI)	*P*	HR (95% CI)	*P*
irAEs				
No				
Yes	0.669 (0.498–0.900)	0.008	0.606 (0.444–0.827)	0.002
ECOG scores				
0–1				
≥ 2	2.700 (1.528–4.770)	0.001	1.885 (1.061–3.347)	0.031
Liver metastasis				
No				
Yes	5.351 (3.976–7.202)	< 0.001	6.110 (3.354–11.131)	< 0.001
Hypertension				
No				
Yes	0.594 (0.418–0.844)	0.004	0.680 (0.474–0.973)	0.035
ALT ratio (2 months after medication/1 week before medication)				
Both normal or ratio ≤ 0.75				
Ratio > 0.75	0.621 (0.434–0.888)	0.009		
AST ratio (2 months after medication/1 week before medication)				
Both normal or ratio ≤ 0.75				
Ratio > 0.75	0.546 (0.380–0.786)	0.001	0.438 (0.302–0.636)	< 0.001

#### Analysis of patient prognostic PFS-related influencing factors

The case characteristic variables in Table 1 were added to the univariate COX regression, which showed that ECOG score ≥ 2 (HR: 2.567, 95% CI 1.458–4.520), having liver metastasis (HR: 3.540, 95% CI 2.578–4.618), VEGFR2 positivity (HR: 0.599, 95% CI 0.360–0.996), and AST (2 months after medication/1 week before medication) > 0.75 (HR:0.626, 95% CI 0.437–0.896) had a lower risk of progression as compared to the control group.

Variables with *P* values below 0.05 for univariate tests and clinical variables of interest irAEs were attempted to be included in multivariate regression analyses corrected for other factors with liver metastasis (HR: 3.031, 95% CI 1.755–5.236), ECOG scores ≥ 2 (HR: 3.585, 95% CI 1.195–10.758) had a higher risk of progression than controls. The risk of progression was higher in the control group and still negatively associated with prolongation of PFS, and the risk of progression was lower in VEGFR2-positive (HR: 0.537, 95% CI 0.313–0.921) than in the control group and still positively associated with prolongation of PFS. The occurrence of irAEs was not associated with PFS (Table 6).

**Table 6:** Univariate and Multivariate COX regression analysis of PFS

PFS	Univariate Cox analysis		Multivariate Cox analysis	
Clinical variables	HR (95% CI)	*P*	HR (95% CI)	*P*
irAEs				
No				
Yes	0.834 (0.621–1.119)	0.227	0.742 (0.423–1.303)	0.299
ECOG scores				
0–1				
≥ 2	2.567 (1.458–4.520)	0.001	3.585 (1.195–10.758)	0.023
Liver metastasis				
No				
Yes	3.540 (2.578–4.618)	< 0.001	3.031 (1.755–5.236)	< 0.001
VEGFR2				
Negative				
Positive	0.599 (0.360–0.996)	0.048	0.537 (0.313–0.921)	0.024
AST ratio (2 months after medication/1 week before medication)				
Both normal or ratio ≤ 0.75				
Ratio > 0.75	0.626 (0.437–0.896)	0.011	0.640 (0.303–1.353)	0.243

## Discussion

Immunotherapy can produce unique inflammatory toxicity while activating the immune system, which is called immune-related adverse effects (Yu et al. 2021). The incidence of irAEs has been reported in the literature to be 55%-60% (Wang et al. 2018), and the incidence of irAEs of different types and degrees occurred in 191 (47.51%) patients in the present study, which is not significantly different from that reported in the literature. In the CheckMate 649 study, the nivolumab combination chemotherapy group for advanced gastric cancer/gastroesophageal junction cancer/esophageal adenocarcinoma showed the most common adverse reactions were nausea (39%), diarrhea (28%), peripheral neuropathy (24%), and vomiting (23%) (Janjigian et al. 2021) In the phase I study, NCT02937116 trial, sintilimab in combination with CapeOx for the treatment of adenocarcinoma of the gastric/gastroesophageal junction produced the main adverse reactions associated with rash (25%) and hypothyroidism (30%) (Jiang et al. 2020), and in the present study were immune-related skin toxicity (23.9%), immune-related endocrine toxicity (23.4%), and immune-related gastrointestinal toxicity (19.9%) was higher in this study, and the main manifestation of immune-related skin toxicity was rash, immune-related endocrine toxicity was hypothyroidism, and immune-related gastrointestinal toxicity was diarrhea, which was not much different from that reported in literature. In clinical work, we will focus on common adverse reactions such as diarrhea, vomiting, and other gastrointestinal reactions, while rare adverse reactions such as neurological toxicity and cardiotoxicity are mainly judged based on the laboratory tests and examination indicators. Some of the safety differences may be related to various factors such as the quality of retrospective data, sample size, physical condition of patients, dosage of medication, and combination therapy programs. There was relevant literature about ICIs-related irAEs reported a median onset time of 42 days (Chen et al. 2021), and the median onset time of toxicity of irAEs in the present study was 49 days, with which the results of the present study are generally consistent. The immunotoxicity in this study was mainly concentrated in grades 1–2, similar to that reported in the literature (Pauken et al. 2019; Postow et al. 2018). In the real world, irAEs are largely relieved with relevant treatment and symptoms. The occurrence of different PD-1 irAEs varies, and a larger sample is needed for analysis.

Studies have demonstrated that the sequence of targeted therapy versus immunotherapy is associated with the incidence of irAEs: one study included 126 NSCLC patients with EGFR mutations who were treated with ICI and EGFR TKIs. Treatment with ICI followed by EGFR TKIs was associated with the emergence of irAEs, but treatment with EGFR TKIs before ICI was not related to the emergence of irAEs (Schoenfeld et al. 2019). While in this paper, prior targeted therapy (OR: 4.360, 95% CI 1.692–11.238) was a risk factor influencing the occurrence of irAEs, the incidence of irAEs was higher in patients with advanced gastric cancer who had received prior targeted therapy before receiving immunotherapy. We already know that targeted therapy sequenced with immunotherapy not only has an impact on efficacy (one study showed that the addition of immunotherapy to targeted therapy improved the prognostic outcome of patients with HER2-positive gastric cancer (Killock 2022). It may also have an impact on the emergence of irAEs. This result is associated with the study with less literature and also needs to do prospective experiments to support it.

In this study, we analyzed the association between the occurrence of irAEs and hypertension. Subgroup analyses showed that the presence or absence of hypertension in oncology patients had a significant between-group difference in the occurrence of irAEs (*P* = 0.008), and multifactorial logistic regression analyses further revealed a higher risk for the occurrence of irAEs in patients suffering from hypertension (OR: 2.759, 95% CI 1.206-6.310), indicating a positive correlation between hypertension and the occurrence of irAEs. In addition, univariate COX regression analysis in hypertension and patients’ prognosis pointed out that patients suffering from hypertension had a lower risk of death as compared to controls (HR: 0.594, 95% CI 0.418–0.844). And multifactorial COX regression analysis showed that hypertension was an independent risk factor for the prognosis of patients with gastric cancer, in which patients with hypertension had a lower risk of death than the control group (HR: 0.680, 95% CI 0.474–0.973), and these differences were statistically significant (*P*-values were all less than 0.05). In another metabolic disease, diabetes, a significant between-group difference in the occurrence of irAEs was similarly found. Although there is no direct evidence in the literature we reviewed that elucidates the relationship between hypertension, diabetes, and immune efficacy, there are studies that point to the fact that hypertension and diabetes do have an impact on the immune system. For example, certain studies have revealed interactions between hypertension, cytokines, and T cells (Guzik et al. 2007; Singh et al. 2014). In addition, some studies have shown that the combination of antihypertensive drugs and immune checkpoint blockers can enhance tumor response and prolong survival in mouse models of breast cancer. This is because cancer cells, in order to survive and proliferate in the body, construct a microenvironment conducive to their growth by compressing blood vessels, altering the body’s pH, and creating a hypoxic environment, which in turn suppresses the function of immune cells. Antihypertensive drugs have the potential to destroy this high-pressure environment created by cancer cells (Chauhan et al. 2019). Also, it has been demonstrated that oncology patients with hypertension are more prone to cardiac irAEs (Chennamadhavuni et al. 2022), while hyperglycemic states similarly affect immune cell function (Daryabor et al. 2020). A study in patients with type 2 diabetes (as compared to nondiabetic patients) also found that PD-1 expression was reduced on peripheral T cells in patients with type 2 diabetes, which may affect the effectiveness of immune checkpoint inhibitors (Sun et al. 2019). In addition, drugs used to treat metabolic diseases, such as metformin, have been shown to have anti-inflammatory effects (Cameron et al. 2016). These findings suggest that hypertension and diabetes may affect the expression of irAEs and the efficacy of immunotherapy. Although these data provide some theoretical support, more prospective studies are needed to further validate these preliminary observations in order to determine the exact relationship between these chronic diseases and immunological efficacy.

Several studies have shown that immunotherapy has become a new option for the treatment of advanced gastric cancer. A CheckMate 649-based study evaluated the efficacy of nivolumab in combination with chemotherapy versus chemotherapy alone in gastric and esophageal adenocarcinomas and showed a 44% reduction in the risk of death in the nabumab-combination-chemotherapy group as compared to the chemotherapy group in patients with a PD-L1 CPS ≥ 5. In all randomized patients, the risk of death was reduced by 38% in the nivolumab combination chemotherapy group (mOS: 14.3 months vs.10.3 months; HR: 0.620) (Janjigian et al. 2018). An ORIENT-16-based assessment of the efficacy of the sintilimab combination chemotherapy group versus chemotherapy alone in patients with locally advanced or metastatic gastro-gastric/gastro-esophageal junction adenocarcinoma demonstrated a significant reduction in the risk of death by 38% in the sintilimab combination chemotherapy group (mOS) for all patients (15.2 months vs. 12.3 months; HR: 0.766); and a reduction in the risk of mortality by 38% in the sintilimab combination chemotherapy group (mOS: 14.3 months vs. 10.3 months; HR: 0.620) in the sintilimab combination chemotherapy group (mPFS: 7.1 months vs. 5.7 months; HR: 0.636) (Xu et al. 2021). In addition, there is the ATTRACTION 4 trial, an Asian population study in patients with HER2-negative, advanced, or recurrent gastric/esophagogastric union cancer, which was conducted in Japan, South Korea, and Taiwan, China. The trial evaluated the efficacy and safety of nivolumab in combination with chemotherapy (SOX or XELOX) versus chemotherapy alone as a first-line treatment. The results showed that first-line treatment with nivolumab in combination with chemotherapy had a mPFS of 10.5 months and a mOS of 17.5 months (Kang et al. 2022). One study summarized the survival time of pembrolizumab monotherapy in Japanese patients, in which, in KEYNOTE-062, the mOS was 20 months in the pembrolizumab group and 18 months in the chemotherapy group (HR: 0.760, 95% CI 0.43–1.33). mPFS (pembrolizumab group vs. chemotherapy group) was 6 vs 7 months (HR: 1.030, 95% CI 0.61–1.74); ORR was 29% vs 34%, respectively (Muro et al. 2023). In our study, the mPFS in the immunotherapy first-line irAEs group was 188 days (95% CI 136.193–239.807), and the mOS was 605 days (95% CI 516.492–693.510). The mPFS in the immunotherapy first-line non-iraes group was 152 days (95% CI 111.733–192.227), and the mOS was 416 days (95% CI 357.866–474.136). The overall immunotherapy efficacy of mOS was 572 days (95% CI 474.71–579.290) and mPFS was 159 days (131.711–186.289). In our study, OS was within the range of prognostic outcomes of immunotherapy. The study design was based on the patient population that met the standard entry criteria to ensure the reliability and comparability of the results. However, there may be some variability in OS due to the practicalities of individualized combination therapy (e.g., in the real world, immunotherapy is often used in conjunction with chemotherapy, targeted therapy, and radiation), as well as differences in clinical patient status and subgroup analysis of clinical patients.

Clinical data shows that immunologic efficacy is reduced in patients with liver metastases (Lee et al. 2022), and Tumeh et al. (2015) first observed a weaker response to PD-1 blockade in patients with liver metastases in 2015. In our clinical work, we have observed that ICIs are less effective in patients with liver metastases. In the present study, we found that patients with liver metastases exhibited a trend toward progression with an elevated risk of death (OS: HR: 6.110, 95% CI 3.354–11.131) (PFS: HR: 3.031, 95% CI 1.755–5.236), while there was little correlation with other metastatic pathways situations.

It has been demonstrated that the higher the ECOG score, the worse the physical status of the patients and the worse the efficacy of immunotherapy (Dall'Olio et al. 2020); our study found that ECOG score ≥ 2 (HR: 1.885, 95% CI 1.061–3.347) The higher risk of death when compared with the control group was still negatively correlated with the prolongation of OS also confirmed this view. Another relevant experimental study demonstrated that VEGFR-2 antibody treatment led to intratumoral immunomodulation and enhanced the antitumor efficacy of PD-L1 blockers in a homozygous mouse tumor model (Li et al. 2022). Our study found that VEGFR2-positive (HR: 0.537, 95% CI 0.313–0.921) had a lower risk of progression as compared to controls, which was still positively associated with prolonged PFS, possibly due to patients receiving VEGFR-2 antibody therapy.

A positive correlation between irAEs and the development of ORR, PFS, and OS in patients treated with ICIs has been reported in the literature (Hussaini et al. 2021; Zhao et al. 2022). The results of this paper showed that the occurrence of irAEs was associated with patients’ OS. The occurrence of irAEs could prolong patients’ OS. Since most of the real-world patients with advanced gastric cancer were treated with first-line therapy, patients’ overall immunologic efficacy was poor, and the sample size of patients treated with backline therapy was small, we did not observe that the occurrence of irAEs was associated with patients’ PFS. Exploring the correlation between irAEs and disease prognosis may be because one of the main reasons for the occurrence of irAEs in ICIs-treated patients is the similarity between antigens presented by tumor cells and normal cells (Hasan Ali et al. 2016), and antigen sharing or cross-reactivity may lead to T cell-mediated responses not only to tumor cells but also to normal cells, and the activated immune system may also target these nontumor sites (Hasan Ali et al. 2016). However, the occurrence of fewer irAEs may lack clinical efficacy, and the occurrence of more irAEs may predict clinical efficacy, which may also create new dilemmas for clinical treatment, and the monitoring of irAEs, as well as their timely treatment, becomes more critical.

The mechanism of the occurrence of irAEs needs to be further investigated by researchers, and whether the occurrence of irAEs can be used as an alternative marker for ICIs needs to be verified in prospective experiments. irAEs treatment is still relatively unitary, and although the safety of irAEs is controllable, serious irAEs can be life-threatening, so the clinical practice needs to manage and control irAEs promptly. Targeting TGF-β and PD-L1 with a bispecific antibody for synergistic cancer immunotherapy. These modified antibodies have shown strong antitumor effectiveness in preclinical and clinical trials, outperforming anti-PD-1/PD-L1 monotherapies (Niu et al. 2023). Recently, Merck announced the discovery of M7824, a bifunctional antibody that suppresses TGF-β and PD-L1 at the same time (Lan et al. 2018). M7824 combines the PD-L1 antibody with a trap structure targeting TGF-β, acting as a neutralizing receptor for TGF-β. Phase I clinical studies show that M7824 therapy has tolerable side effects and has shown therapeutic efficacy in a variety of cancer types (Strauss et al. 2018). Later, other bispecific antibodies (BsAbs) are created; they include YM101 and BiTP, which show strong antitumor effects in preclinical and clinical research (Yi et al. 2022, 2021). Further exploratory analysis can also be conducted on this in the future.

## Conclusions

In summary, the incidence of irAEs in the real world is still relatively common, but the safety can be controlled with treatment. By retrospectively analyzing the data of patients with advanced gastric cancer treated with ICIs previously admitted to our medical center, we found some clinical characteristic factors associated with the occurrence of irAEs as well as the efficacy and prognosis: the presence or absence of hypertension, whether or not to receive targeted therapies can predict the occurrence of irAEs, and the more the presence of irAEs, the better the prognosis. These can help clinicians in clinical drug selection. To obtain more accurate data, it is necessary to expand the sample size further or conduct prospective experiments.

## Supplementary Information

Below is the link to the electronic supplementary material.

Supplementary file 1 (DOCX 17 KB)

## Data Availability

The data presented in this study can be made available, on request, by the corresponding author.
