# Supplementary material for: Safety and clinical efficacy of immune checkpoint inhibitors in advanced gastric cancer in the real world
Source: J Cancer Res Clin Oncol. 2024 Apr 8;150(4):180. doi: 10.1007/s00432-024-05703-8 (PMC11001672; doi:10.1007/s00432-024-05703-8)
Supplement: Supplementary file 1 — (DOCX 17 KB) [file 432_2024_5703_MOESM1_ESM.docx]

Supplementary Table 1: Detailed information on the patient's medication. (supplementary material)

| Medication regimen | Medicine | Dosage | Administration | Medication cycle | Mode of administration |
| --- | --- | --- | --- | --- | --- |
| PD-1 monoclonal antibodies | Pembrolizumab | 200 mg | d1 | Repeat every 21 days | i.v.gtt |
|  | Sintilimab | weight <60 kg: 3 mg/kg; >60 kg: 200 mg fixed-dose | d1 | Repeat every 21 days | i.v.gtt |
|  | Toripalimab | 240 mg | d1 | Repeat every 21 days | i.v.gtt |
|  | Tislelizumab | 200 mg | d1 | Repeat every 21 days | i.v.gtt |
|  | Nivolumab | 3 mg/kg | d1 | Repeat every 14 days | i.v.gtt |
|  | Camrelizumab | 200 mg | d1 | Repeat every 21 days | i.v.gtt |
|  | Penpulimab | 200 mg | d1 | Repeat every 14/21 days | i.v.gtt |
| First-line regimen: PD-1 combined with CAPOX or SOX regimens | Oxaliplatin | 130 mg/m^2^ | d1 | Repeat every 21 days | i.v.gtt |
|  | Capecitabine | 1000 mg/m^2^ | b.i.d. | On days 1-14 every 3 weeks | p.o. |
|  | S-1 | BSA <1.25 m^2^: 40 mg/time; 1.25~1.5 m^2^: 50 mg/time; >1.5 m^2^: 60 mg/time | b.i.d. | On days 1-14 every 3 weeks | p.o. |
| Second-line regimen: PD-1 in combination with paclitaxel analogs or camptothecin analogs | Paclitaxel analogs | | | | |
|  | Albumin-bound paclitaxel | 100 mg/ m^2^ | d1, d8, d15 | Repeat every 28 days | i.v.gtt |
|  | Docetaxel | 75-100 mg/m^2^ | d1 | Repeat every 21 days | i.v.gtt |
|  | Camptothecin analogs | | | | |
|  | Irinotecan | 150~180 mg/m^2^ | d1 | Repeat every 14 days | i.v.gtt |
| Third-line regimen: PD-1 in combination with apatinib or other targeted agents | Apatinib | 200 mg-500 mg | q.d. | Repeat every 28days | p.o. |
|  | Anlotinib | 12 mg/time | q.d. | On days 1-14 every 3 weeks | p.o. |
|  | Bevacizumab | 7.5 mg/kg | d1 | Repeat every 21 days | i.v.gtt |
|  | Lenvatinib | weight < 60 kg, 8 mg; ≥ 60 kg, 12 mg | q.d. | Continued treatment until intolerable toxic effects | p.o. |
